# Compensatory and Catalyzing Beliefs: Their Relationship to Pro-environmental Behavior and Behavioral Spillover in Seven Countries

**DOI:** 10.3389/fpsyg.2019.00963

**Published:** 2019-05-21

**Authors:** Stuart Capstick, Lorraine Whitmarsh, Nick Nash, Paul Haggar, Josh Lord

**Affiliations:** School of Psychology and Tyndall Centre for Climate Change Research, Cardiff University, Cardiff, United Kingdom

**Keywords:** behavioral spillover, compensatory beliefs, pro-environmental behavior, pro-environmental identity, survey methods

## Abstract

There is growing research interest in behavioral spillover and its potential for enabling more widespread lifestyle change than has typically been achieved through discrete behavioral interventions. There are some routes by which spillover could take place without conscious attention or explicit recognition of the connections between separate behaviors. However, in many cases there is an expectation that an individual will perceive behaviors to be conceptually related, specifically in terms of their compensatory (suppressing further action) or catalyzing (promoting further action) properties, as a prerequisite for both negative and positive spillover. Despite this, relatively little research has been carried out to assess the beliefs that may underpin spillover processes as held by individuals themselves, or to measure these directly. We develop and evaluate a survey-based instrument for this purpose, doing so in a sample of seven countries worldwide: Brazil, China, Denmark, India, Poland, South Africa, and the United Kingdom (approx. 1,000 respondents per country). This approach allows us to assess these measures and to compare findings between countries. As part of this, we consider the connections between beliefs about behavioral relationships, and other key variables such as pro-environmental identity and personal preferences. We observe higher levels of endorsement of compensatory beliefs than previous research, and even higher levels of endorsement of novel items assessing catalyzing beliefs. For the first time, we present evidence of the validity of such measures with respect to comparable constructs, and in relation to people’s consistency across different types of behaviors. We reflect on the implications of considering the relationships between behaviors in the context of people’s subjective beliefs and offer recommendations for developing this line of research in the broader context of spillover research and within a cross-cultural framework.

## Introduction

Recent years have seen a growth in research that has set out to promote, understand, and test behavioral spillover in the environmental domain. Behavioral spillover is broadly defined as an observable and causal effect one behavior has on another (Nash et al., 2017). Research in this area has been founded on an appreciation of the limited capacity for piecemeal behavior change to address urgent environmental problems (Maniates, 2001), especially through simple, low-effort individual action (Thøgersen and Crompton, 2009). The prospect that such behaviors might nevertheless prompt or catalyze more widespread behavior change has generated interest in the relationship between environmentally significant behaviors, and the conditions under which one action might “spill over” to another (Defra, 2008). Similarly, evidence that interventions to promote pro-environmental behavior (PEB) may be undermined by rebound effects (e.g., installing domestic insulation leading to greater energy use) highlights a need to understand how and why these apparently inconsistent behaviors may occur and ultimately to reduce their occurrence.

While there are various proposed mechanisms for how spillover works, most assume that they require some degree of conscious reflection – for example, justifying inconsistent behaviors (e.g., eating cake after exercising) or motivating consistent ones (e.g., giving money to charity leading to volunteering). Yet, while patterns of compensatory and catalyzing behaviors have been explored – a central objective of spillover research – individuals’ own beliefs about these behaviors have received relatively lesser attention. In the present study, we consider how compensatory and catalyzing beliefs relate to PEBs, as well as to underlying psychological constructs. In order to examine these beliefs in light of the types of behavioral patterns that would be anticipated as a result of spillover processes, we also examine whether and how they are linked to consistency across self-reported behaviors.

## Spillover Mechanisms and the Role of Beliefs About Behavior

Recent reviews focusing specifically on spillover of pro-environmental behavior have highlighted several mechanisms by which the process might occur, as well as different perspectives on what is encompassed or excluded from the concept of spillover itself (Truelove et al., 2014; Nash et al., 2017; Nilsson et al., 2017). While there is the potential for spillover to occur automatically or outside of a person’s awareness, much research indicates that conscious emotional, self-perception, or mental accounting processes are activated in triggering spillover. The types of conscious reasoning and justifications typical to spillover are neatly articulated by Dolan and Galizzi’s (2015) explanation of the processes by which one type of healthy or unhealthy behavior (running or sofa-sitting) can lead to another (eating more or less healthily). The concept of “*promoting*” (positive) spillover occurs when behaviors work together; for example, “*I ran an hour, let’s keep up the good work.*” In “*permitting*” (negative) spillover, behaviors work against each other; for example, “*I ran an hour, I deserve a big slice of cake.*” Similarly, permitting spillover might also be triggered by the sofa-sitter concluding that “*I’ve been lazy today, let’s have a big slice of cake.*” “Purging” spillover (moral cleansing) occurs when an actor attempts to reduce negative feelings after indulging, taking the view that “*I’ve been lazy today, best not eat so much tonight.*”

Of particular relevance to the present study, Nash et al. (2017) point to the potential for self-perception to underpin spillover: the idea that reflecting on past behavior provides cues to people for how to act subsequently. In a related manner, though drawing on a different strand of theory, it has also been argued that spillover may be underpinned by people’s desire for consistency in their actions and with their values, not least because the perception of inconsistency – or dissonance – can be psychologically uncomfortable (Sapiains et al., 2015).

While people’s awareness of the links between behaviors can promote positive spillover (i.e., one “good” behavior leading to another) equivalent processes may operate that could undermine this, or operate in a reverse manner. For example, Nilsson et al. (2017) outline in some detail the types of reasoning or rationalization that might underpin negative spillover, with the notion of “moral licensing” held to be central. This entails a person balancing the “good” of one action with the “bad” of another: having carried out one PEB they may consider that they have earned the right (or “licence”) to act in another, less pro-environmental manner (Khan and Dhar, 2006; Merritt et al., 2010; Blanken et al., 2015); or they may simply be of the view that having now done their share, they have reduced their obligation to take further action.

## The Role of Psychological and Cultural Factors as Influences on Spillover and Behavioral Consistency

Pro-environmental action is influenced by a range of factors including people’s values, general beliefs, and identity (Hornsey et al., 2016). In relation to spillover in particular, as well as consistency across behaviors, a person’s pro-environmental identity has been argued to be critical. From a theoretical perspective, it is a person’s “self-identity” – their concept of themselves – that is used to guide actions. In the environmental domain, this manner of self-identity has been shown both to be a significant predictor of PEB (Sparks and Shepherd, 1992), and been proposed as a factor that promotes behavioral consistency (Whitmarsh and O’Neill, 2010). Several studies have furthermore assumed a central role for pro-environmental identity in enabling spillover processes. For example, experimental work has shown that drawing attention to the environmental impacts of choices can lead to a heightened sense of one’s pro-environmental self-identity, which in turn can promote subsequent actions in line with this self-perception (Cornelissen et al., 2008; Poortinga et al., 2013; Van der Werff et al., 2013; though see Truelove et al., 2016, for contradictory findings). More generally, research has shown that the potential exists for people to evaluate their behaviors in the context of their identity: for example, Gneezy et al. (2012) argue that high-cost behaviors in particular may be perceived by a person to reflect a pro-social identity, and consequently to raise the likelihood of further pro-social action. Given the centrality of identity to spillover research, and to PEB more generally, we seek to understand its relationship to the types of beliefs that are a focus of the present study. We conceptualize pro-environmental identity in terms of the self-concept, which stresses how a person sees themselves, in the context of their environmental concerns and behaviors. In this we draw on research by Whitmarsh and O’Neill (2010) who developed the identity scale we apply in the present study.

Although less considered in the environmental psychology literature, a separate strand of research has also highlighted how people’s preference for consistency (PFC) is related to patterns of behavior. PFC refers to the idea that people value behavioral characteristics that are stable, predictable, and reliable (Guadagno and Cialdini, 2010). Whereas more general theoretical frameworks have tended to assume by default that people are motivated to be consistent to an equivalent degree, the PFC framework proposes that, instead, there are individual differences in the extent to which people’s actions are congruent with past and similar behavior (Guadagno and Cialdini, 2010). For example, Cialdini et al. (1995) found that PFC moderated how susceptible people were to the “foot-in-the-door” effect, in which the request to carry out a small action allows for a subsequent, larger request to be met; this effect has itself been used often as an analog of spillover (Nash et al., 2017). Given the demonstrated utility of PFC as a construct that underpins behavioral consistency in general terms, we are interested to understand the extent to which it is related to the types of beliefs considered in the present study.

As we outline above, there is evidence for individual differences in behavioral consistency and PFC. In a related manner, cross-cultural research has indicated that there are differences in the extent to which societies tolerate ambiguity; this has been linked to cultural variability in uncertainty avoidance (the degree to which a society challenges or is accepting of unpredictability: Hofstede, 2011). Variation in tolerance of ambiguity, in turn, has direct implications for how a person’s underlying values influence their behavior (Furnham and Ribchester, 1995; Boer and Fischer, 2013). In particular, and in a manner analogous to the individual-level need to manage cognitive dissonance, individual and societal differences in this area may affect the extent to which people accept and manage personal (in)consistency (Boer and Fischer, 2013).

In line with the expectation that behavioral consistency – and by implication, spillover processes – is likely to vary across cultures and countries, in the present study we assess the endorsement, and implications, of compensatory and catalyzing beliefs across several different nations, including non-Western contexts. This builds on prior work which has addressed spillover in research primarily carried out in Europe and North America, as well as on prior work examining pro-environmental behaviour across nations and cultures (Oreg and Katz-Gerro, 2006). Given the almost complete absence of cross-national comparative work on spillover in general – and the role of underlying beliefs in particular – we are interested to ascertain the extent to which our findings are obtained consistently across countries.

## Measurement and Predictive Ability of Compensatory and Catalyzing Beliefs

Despite conceptual and theoretical reasons to expect that the types of catalyzing and compensatory beliefs outlined above might be related to a person’s PEB, there has been surprisingly little research that has directly addressed this.

One study that did set out to assess beliefs of this kind was work by Kaklamanou et al. (2015), who devised a 16-item measure of “compensatory green beliefs.” This was designed to assess the extent to which people endorsed beliefs about one type of PEB compensating for another. As these authors pointed out, such compensatory beliefs have been more widely considered in the health domain, with some research finding a relationship with health risk behaviors and dietary temptations (Knäuper et al., 2004; Albarracin et al., 2009). Indeed, these and other studies have found compensatory health beliefs are related to intentions to quit smoking (Radtke et al., 2011) and other health risk behaviors such as drinking alcohol and unhealthy eating (Knäuper et al., 2004).

The compensatory beliefs scale devised by Kaklamanou et al. (2015) covered a range of behaviors and posited relationships between them. For example, items included the proposition that “If you have a low flush toilet, then it is okay to use more water in other ways” and “Composting food waste can make up for buying imported food,” each referring to trade-offs within domains (water and food, respectively). Behavior pairs were also proposed that were cross-domain, such as “Walking to the supermarket can compensate for buying highly packaged food” and “Having a water butt can compensate for using the oven.”

The study by Kaklamanou et al. (2015) found that the compensatory beliefs scale was negatively associated with ecological worldview and pro-environmental identity; and that the scale also negatively predicted self-reported PEB over and above these variables. This suggests these beliefs tended to be connected to relatively less pro-environmental views and actions, in line with the exculpatory tone of the phrasing used. For the most part, the items used tended to have low levels of agreement. In all but five cases, participant agreement with the statements presented was lower than 10%, with the highest level of agreement being for a travel-related proposition, “not driving a car compensates for flying on holiday” (16.2% agreement); this particular statement may also have chimed with Barr et al. (2010) finding that holiday-related behaviors were seen as particularly distinct from everyday domestic choices in the home.

Overall, the low levels of agreement found by Kaklamanou et al. (2015) may have reflected that such compensatory green beliefs are relatively uncommon, or that the particular examples used were not endorsed. There is also the possibility that people’s willingness to equate their own views with compensatory beliefs may have been affected by social desirability, whereby such beliefs could be considered disagreeable. Nevertheless, these findings parallel an earlier study by Bratt (1999) in which levels of endorsement of three compensatory statements were also found to be low: indeed, in that study the item presenting a trade-off between not driving and flying on holiday was agreed with by a similar proportion of respondents, at 17.1%.

Building on this earlier work, Byrka and Kaminska (2015) argued that a useful avenue to develop an understanding of compensatory beliefs was to consider them in terms of their relative similarity and difficulty. In particular, these authors suggested that compensatory behaviors are more likely to operate as such if they fall under the same category of behavior (similarity of domain) than if they are dissimilar. As such, it might be expected that an item referring to compensating for buying imported food by composting would be seen as more plausible than compensating for using an oven by using a water butt – to use examples taken from the work by Kaklamanou et al. (2015). Indeed, Byrka and Kaminska (2015) made the argument that across the items developed for that earlier study, the most-endorsed did indeed tend to be those that reflected within-domain trade-offs.

The study by Byrka and Kaminska (2015) proposed, in addition, that behaviors which were easier than the preceding “target” behaviors would be more likely to be endorsed in terms of a compensatory process than would a more difficult choice. For example, the reuse of a carrier bag obtained from a store would be seen as a plausible compensatory act, in part due to its being a simple action to perform; in contrast to using environmentally friendly cleaning products to compensate for using an insecticide. Across their analyses, these authors found that endorsement of compensatory beliefs was higher where target and compensatory behaviors were in the same domain, and where the compensatory behavior was easier than the target behavior.

Other research by Seebauer (2018) has used measures designed to test rebound effects of acquiring an electric car or carrying out home insulation; as well as items that presented these actions in terms of compensatory behaviors for other environmentally significant choices (for example: “I use an electric car, so it doesn’t matter much if I fly on a holiday every now and then”). As in the studies described above, this research found that compensatory beliefs were negatively associated with pro-environmental values. In addition, some evidence was presented that rebound behaviors – for example, reporting that one covered more miles with an electric vehicle than before – were also associated with compensatory beliefs.

In addition to survey-based work that has assessed the prevalence and measurement of compensatory beliefs, recent qualitative research by Hope et al. (2018) has shed light on their nature – as well as the ends to which they might be put. These authors suggest that compensatory beliefs can serve important functions in terms of enabling people to affirm their own environmental credentials (even though they may be aware of other actions that are less desirable), to justify some (harmful) actions, and to reduce their negative feelings about their impact on the environment.

## Aims of the Present Study

The studies considered above have shed light on the prevalence of certain types of belief of relevance to spillover processes and behavioral consistency. However, there are a number of limitations to the research carried out to date that we seek to address.

First, the focus of prior work has been almost exclusively upon people’s justification for *inconsistency* across PEBs. In all cases, the measures described above are framed in terms of a “negative” behavior balanced against a “positive” one – either being presented in terms of negative action that is permitted on account of taking other, positive behavior; or in terms of a positive action compensating for other, negative action (hence, the use of the term “compensatory” beliefs). However, prior work has not reflected the potential for equivalent processes whereby one positive action might give rise to another. In the present study, we therefore develop a new measure of “catalyzing” beliefs, intended to complement this former construct. Our concept of a catalyzing belief is one that views behaviors as positively related, whereby action in one area is understood as a trigger for action in another. Given the conceptual linkages between “compensatory” and “catalyzing” beliefs and spillover, we refer in places to both of these as constituting “spillover-related” beliefs.

Second, previous work has set out to measure compensatory beliefs exclusively in terms of trade-offs between defined PEBs: for example, between use of a car and donating to an environmental organization. Although this approach enables a comparison between types of PEBs, such as similarity and difficulty as in Byrka and Kaminska (2015)’s study, these measures have not allowed for an examination of more generalized compensatory beliefs. In the present study, we build on this prior work through an assessment of more general beliefs about the relationships between behaviors, as well as between specified behavior pairs.

Third, although the measures used to date have been considered in the context of other environmentally significant measures, such as pro-environmental identity, ecological worldview, and personal norms, there has not yet been an attempt to validate scales or items with reference to conceptually related constructs. As well as assessing a link with pro-environmental identity in the present study, we also consider our measures of spillover-related beliefs in relation to Cialdini et al.’s (1995) notion of personal consistency and their PFC scale, in order to address the convergent validity of the scales we present. We consider these relationships separately across countries, and for the dataset as a whole, in order to offer an extension of previous research that has occurred in the context of a single country.

Fourth, while previous work has been able to assess compensatory belief measures in relation to several indicators of PEB, there has to date been no analysis of whether and how the scales and items used actually reflect *relationships* between behaviors. It remains unclear, for example, whether those who endorse compensatory beliefs show related *patterns* of behavior in line with this. We are interested here to assess the linkages between different types of behavior, rather than cross-national differences. For this reason we use aggregated data and analyses from participants across countries to assess this study aim.

Finally, the research assessing these types of beliefs has, to date, been able to do so only in homogenous settings and primarily in European or other “Western” nations. In the present study, we consider the application of measures across diverse cultures, extending our survey research to Brazil, China, India, and South Africa, as well as the European countries of Denmark, Poland, and the United Kingdom. We approach this in an exploratory manner, without a pre-specified hypothesis, in order to characterize similarities or differences in the presence of such beliefs across different national contexts.

Our research questions are as follows:

(1) To what extent are compensatory and catalyzing behavioral beliefs endorsed in different national contexts?
(2) To what extent are compensatory and catalyzing beliefs related to pro-environmental identity and PFC?
(3) To what extent are compensatory and cataylzing beliefs related to self-reported PEB?
(4) To what extent are compensatory and catalyzing beliefs related to consistency across different self-reported PEBs?

Based on previous work which has found correlations between compensatory beliefs, pro-environmental identity, and PEB, we anticipate that the measures used here will demonstrate similar associations. We also offer additional predictions based on further novel components to this study. Our hypotheses are as follows:

H1. Pro-environmental identity will negatively predict compensatory beliefs (H1a), and pro-environmental identity will positively predict catalyzing beliefs (H1b).
H2. Preference for consistency will negatively predict compensatory beliefs (H2a), and preference for consistency will positively predict catalyzing beliefs (H2b).
H3. Compensatory beliefs will negatively predict PEB (H3a), and catalyzing beliefs will positively predict PEB (H3b).
H4. Compensatory beliefs will negatively predict consistency across different behaviors (H4a), and catalyzing beliefs will positively predict consistency across different behaviors (H4b).

Hypotheses H1 and H2 assess aspects of the second research question (links between psychological constructs and spillover-related beliefs). Hypotheses H3 and H4 are derived from research questions 3 and 4, respectively (levels and patterns of self-reported behavior).

## Materials and Methods

### Participants and Design

Participants were recruited through the research panel provider Qualtrics. We used quota sampling in order to ensure the participant pools in each of the surveyed countries were representative by age, gender, and income, based on publicly available national statistics. In selecting for age, we used bands (e.g., 18–24, 25–34, 35–44, etc.) which we matched to national demographics (e.g., in the United Kingdom to that provided by the Office for National Statistics). For all countries, the median age band was 35–44 years of age; with the exception of the United Kingdom and Denmark where this was 45–54 years of age. We quota sampled for personal income, based on a country’s income quintiles, such that the samples obtained reflected a range of income brackets. We sought to obtain a 50:50 split for gender, while allowing respondents to self-identify in another way than male or female. While we did not quota sample for education, this information was obtained through a survey item. There was a reasonable spread of levels of education, although this may have been skewed somewhat toward those with a higher level of education: while it is problematic to compare across countries given different systems, around two-thirds (63%) of the sample had a graduate-level qualification.

Participants completed survey questionnaires online between March and November 2016, receiving a small compensation for participating (credits administered by the panel provider). The median time taken to complete the survey was 29 min 50 s. The full sample of respondents comprised 6,969 individuals, approximately 1,000 people per country surveyed (although due to problems with obtaining a full sample in Poland, numbers were lower here at *n* = 658; in India we obtained a sample *n* = 985, with just over *n* = 1,000 in other countries).

For each of the surveyed countries, items were translated by professional translators, and subsequently double-checked by a second professional translator. In addition, collaborators based in academic institutions in each of the countries surveyed were involved in checking for meaning and transferability to that country’s context.

### Measures

Items were administered in blocks of questions, using the online survey randomization feature to preclude ordering effects.

The survey incorporated a range of measures, not all reported or analyzed here. The following items and scales are those considered in the present study.

#### Compensatory Beliefs

We measured compensatory beliefs using nine items, developed in part to build on earlier work by Kaklamanou et al. (2015). The items were designed to reflect specific behavior pair trade-offs as well as more general compensatory beliefs. Items included statements such as “If I save electricity through switching off appliances, I am entitled to use it in other ways such as by turning up the heating” and “Doing some things that are positive for the environment means I am allowed to do other things that are less environmentally friendly.” Participants were asked the extent to which they agreed or disagreed with each statement, on a scale from “1” (entirely disagree) to “7” (entirely agree). The full list of items is given in Table 1, together with descriptive statistics for overall levels of agreement. The compensatory beliefs items formed a reliable scale in all countries; alpha scores obtained were as follows: Brazil (α = 0.76), China (α = 0.84), Denmark (α = 0.78), India (α = 0.87), Poland (α = 0.73), South Africa (α = 0.81), and United Kingdom (α = 0.86).

**Table 1:** Items and descriptive statistics for compensatory and catalyzing beliefs scales (all countries).

	Compensatory beliefs			

Item	Mean (SD)	% Agree (cross-national)	Highest % agree (country)	Lowest % agree (country)
Doing some things that are positive for the environment means I am allowed to do other things that are less environmentally friendly	3.25 (1.92)	26.2%	42.4% (India)	14.6% (Denmark)
As long as I take a few simple actions to protect the environment then that is enough	4.24 (1.63)	47.6%	67.0% (Poland)	29.8% (Denmark)
I already try to help out on environmental issues; I am not prepared to change my lifestyle any further	3.95 (1.59)	36.7%	45.7% (Poland)	27.9% (South Africa)
If I save electricity through switching off appliances, I am entitled to use it in other ways such as by turning up the heating	3.40 (1.95)	29.4%	51.4% (China)	6.6% (Denmark)
As long as I “do my bit” to help the environment at home, there is no need to worry about doing this at work or in other situations	2.84 (1.75)	18.4%	46.2% (India)	8.5% (Denmark)
The environmental impact of flying on holiday can be made up for by reducing one’s car use at other times	4.13 (1.61)	39.5%	66.8% (India)	16.2% (Denmark)
Reducing my environmental impact at home (e.g., by recycling) helps to compensate for any environmental impacts I have at work or elsewhere	3.92 (1.84)	41.4%	62.2% (India)	26.0% (Denmark)
It doesn’t matter how much energy I use when I’m at work or out of the house, as long as I try to be “green” at home	2.68 (1.69)	15.9%	38.6% (India)	4.9% (Denmark)
If a person has a diet that is environmentally friendly, this compensates for any environmental harm from them burning petrol/diesel in cars	3.03 (1.76)	20.7%	41.7% (India)	3.6% (Denmark)
*Full scale*;	31.43 (10.25)			
*Equivalent per item*	3.49 (1.14)			

	**Catalyzing beliefs**			

Being environmentally friendly is not about taking small actions, it is a complete approach to life	5.43 (1.58)	77.0%	92.8% (China)	59.7% (Denmark)
Doing something positive for the environment in my everyday life makes me want to do other similar things	5.33 (1.28)	76.7%	91.8% (India)	58.8% (Denmark)
If I manage to do one small thing for the environment, it gives me the sense that bigger changes in my lifestyle are possible	5.30 (1.35)	75.9%	91.5% (India)	44.8% (Denmark)
If I act in a manner that benefits the environment, it makes me more aware of other similar actions I can take	5.44 (1.24)	81.8%	93.3% (India)	66.6% (United Kingdom)
*Full scale*;	21.53 (4.22);			
*Equivalent per item*	5.38 (1.06)			

#### Catalyzing Beliefs

We measured what we term “catalyzing” beliefs using four novel items. These were designed to mirror the types of statements used to reflect compensatory beliefs, but in contrast to convey the belief that undertaking positive PEB was associated with taking further action in that vein. The items used in all cases were intended to convey a generalized belief in this catalyzing property of PEB. Items included the statements “Doing something positive for the environment in my everyday life makes me want to do other similar things” and “If I manage to do one small thing for the environment, it gives me the sense that bigger changes in my lifestyle are possible.” Participants were asked the extent to which they agreed or disagreed with each statement, on a scale from “1” (entirely disagree) to “7” (entirely agree). The full list of items is given in Table 1, together with descriptive statistics for overall levels of agreement. The catalyzing beliefs items formed a reliable scale (fair to excellent alpha scores) in all countries; alpha scores obtained were as follows: Brazil (α = 0.71), China (α = 0.77), Denmark (α = 0.66), India (α = 0.71), Poland (α = 0.69), South Africa (α = 0.71), and United Kingdom (α = 0.81).

#### Pro-environmental Identity

Seven items were used to measure pro-environmental identity, using items adapted from previous research (Whitmarsh and O’Neill, 2010) as follows: “Taking action to protect the environment is an important part of who I am,” “I would describe myself as an environmentalist,” “I would not want anyone to think of me as someone who is concerned about reducing waste” (reverse-scored), “I would not want my family or friends to think of me as someone who is concerned about environmental issues” (reverse-scored), “I am the type of person who tries not to be wasteful,” “I think of myself as an environmentally friendly person,” and “I would be embarrassed to be considered a ‘waste-conscious’ person” (reverse-scored). Participants were asked the extent to which they agreed or disagreed with each statement, on a scale from “1” (entirely disagree) to “7” (entirely agree). The pro-environmental identity items formed a fairly reliable scale across countries, though with somewhat lower alpha scores than those obtained for other scales; alpha scores obtained were as follows: Brazil (α = 0.59), China (α = 0.70), Denmark (α = 0.72), India (α = 0.53), Poland (α = 0.58), South Africa (α = 0.65), and United Kingdom (α = 0.75).

#### Preference for Consistency

We used seven items taken or adapted from Cialdini et al.’s (1995) PFC scale, as follows: “It is important to me that my actions are consistent with my beliefs,” “Admirable people are consistent and predictable,” “I get uncomfortable when I find my behaviour contradicts my beliefs,” “I’m uncomfortable holding two beliefs that are inconsistent,” “It doesn’t bother me much if my actions are inconsistent” (reverse-scored), “It is important to me that those who know me can anticipate what I will do,” and “I want to be described by others as a stable, predictable person.” Participants were asked the extent to which they agreed or disagreed with each statement, on a scale from “1” (entirely disagree) to “7” (entirely agree). The PFC items formed a reliable scale in all countries; alpha scores obtained were as follows: Brazil (α = 0.64), China (α = 0.62), Denmark (α = 0.72), India (α = 0.62), Poland (α = 0.70), South Africa (α = 0.70), and United Kingdom (α = 0.77).

#### Pro-environmental Behavior

We used a battery of 20 items designed to measure self-reported incidence of carrying out a range of PEBs. These items were derived in part from previous studies of PEBs (e.g., Whitmarsh and O’Neill, 2010) and from qualitative research previously carried out in six of seven of the surveyed countries (Nash et al., under review). Participants were asked to state the frequency with which they had carried out these behaviors, on a scale from “0” (not at all in the past year) to “10” (at least once a day). The full list of items is given in Table 2, together with descriptive statistics. PEBs included in the battery include those relating to “private-sphere” (i.e., consumer or domestic) action (see Stern, 2000), including “avoided wasting food (e.g., by using leftovers)” and “bought environmentally friendly products” as well as “public-sphere” (i.e., political or social) action, including “encouraged other people to save energy” and “donated money to an environmental campaign group.” Due to ethical and practical considerations, Chinese respondents were asked four items in substitution for the more politically sensitive items.

**Table 2:** Component structure and descriptive statistics of PEBs across six countries (Brazil, Denmark, India, Poland, South Africa, and United Kingdom).

	Component/Factor			
	1	2	3	Mean (SD)
Took part in a protest about an environmental issue	**0.807**	−0.068	0.141	0.84 (1.83)
Got involved in conservation work to protect natural environments (e.g., national parks, coastline)	**0.802**	0.059	0.162	1.43 (2.37)
Offered support (e.g., by voting) for political action to protect the environment	**0.797**	0.047	0.142	2.40 (2.24)
Contacted a politician about an environmental issue	**0.779**	−0.105	0.091	0.66 (1.64)
Signed a petition about an environmental issue	**0.770**	0.041	0.111	1.37 (2.17)
Donated money to an environmental campaign group	**0.741**	−0.022	0.207	1.16 (1.88)
Done something together with neighbors, people at work or friends to address an environmental issue	**0.686**	0.081	0.301	1.77 (2.40)
Found out more about environmental issues (e.g., learning more about climate change)	**0.575**	0.244	0.344	3.33 (2.71)
Avoided buying new things (e.g., clothes, luxury items)	0.338	0.269	0.237	3.60 (2.79)
Avoided wasting food (e.g., by using leftovers)	0.054	**0.712**	0.129	7.36 (2.09)
Avoided littering (throwing rubbish on the street)	−0.116	**0.684**	−0.015	8.10 (1.92)
Turned off the tap when brushing teeth	−0.028	**0.669**	0.061	8.07 (2.21)
Turned off lights when not in use	−0.140	**0.630**	0.130	8.25 (1.76)
Taken short showers (less than 3 min long) or infrequent baths	0.172	**0.536**	0.182	6.12 (3.08)
Recycled household waste (e.g., glass, plastic, food waste)	0.134	**0.408**	0.161	5.82 (3.14)
Encouraged other people to save energy	0.378	0.394	0.339	4.40 (2.93)
Eaten organic, locally grown or in season food	0.131	0.160	**0.787**	4.96 (2.80)
Bought environmentally friendly products	0.227	0.158	**0.769**	4.50 (2.48)
Bought products with less packaging	0.229	0.261	**0.694**	4.50 (2.59)
Avoided eating meat	0.204	0.066	**0.529**	3.10 (3.29)

In order to assess the latent structure – and hence behavior “types” – across the PEB items, we carried out a principal components analysis. Given the use of several alternative or modified items in the China survey (e.g., relating to “voting” or “protest”), we carried out this analysis on data from the remaining six countries: Brazil, Denmark, India, Poland, South Africa, and the United Kingdom. Principal components analysis was undertaken based on eigenvalues >1 and using Varimax rotation. We used a Varimax (orthogonal) rotation in order to derive distinct (uncorrelated) principal components; this enables us to compare consistency across different types of PEB, as we describe below. An alternative approach using oblique rotation (in which principal components are permitted to correlate) reveals a similar latent structure to that described below. We did not apply this approach, however, given our particular interest in the extent to which people varied in their consistency across different types of behavior; we consider it would have been problematic to calculate differences between factor scores – our approach to operationalizing “consistency” – had those factors been known to be substantially correlated.

The factor structure of the PEB items for the six-country dataset is shown in Table 2, with factor loadings above 0.4 shown in bold. The types of PEB obtained fall under three fairly neatly delineated categories. Factor 1 encompasses public-sphere behavior (e.g., signing a petition, donating money) incorporating one behavior relating to finding out more about climate change; factor 2 encompasses resource-use and waste-avoidance behaviors, including limiting water and energy usage, as well as recycling; factor 3 encompasses purchasing as well as food-related behaviors (e.g., buying environmentally-friendly products). For subsequent analyses we name the factors accordingly. As we discuss below, we use factor scores in our analyses; however, we also note that measures of alpha corresponding to each of the three factors indicate acceptable to excellent reliability (assuming items with loadings >0.4, factor 1 α = 0.90, factor 2 α = 0.66, factor 3 α = 0.74).

#### Analytic Approach

We adopt several, related approaches in order to address the study’s research questions and hypotheses. In the first instance, we describe the distributional properties of the compensatory and catalyzing beliefs scales. This enables us to compare the extent to which they are endorsed across the seven countries. Next, we carry out correlation analyses to assess the extent to which pro-environmental identity is predictive of compensatory and catalyzing beliefs. We examine the relationship between these beliefs and PFC in a similar manner.

In order to consider the relationships between the spillover-related constructs and PEB, we first assess the extent to which compensatory and catalyzing beliefs relate to different types of PEB, based on the factor analysis of behaviors. Having done so, we then examine consistency *between* behavior types and whether this is related to compensatory and catalyzing beliefs.

We adopt the approach of using factor scores for each of the three principal components (factors), which in each case represents a score weighted to reflect the relative loading of items within the factor. In this, we follow the “weighted sum scores” approach described by DiStefano et al. (2009). The use of factor scores enables us to obtain a participant score for each behavior type, which can be treated as an outcome variable in linear regression analyses.

In order to develop an indicator of consistency between behavior types, we calculate the positive difference between factor scores for each participant, across the three factors. For example, to calculate the difference between factors 2 and 3, we use the following equation, where *D* is the positive value of the difference between the two factor scores and where FAC2 and FAC3 represent scores for factors 2 and 3:

$$\text{D}=\surd ({\left(\text{FAC}2-\text{FAC}3\right)}^{2})$$

This enables us to quantify the extent to which each participant’s PEB is relatively consistent across behavior types (a small positive value for *D*) or relatively inconsistent (a large positive value for *D*). We carry out this assessment of difference for each of the pairs of factors (i.e., factor 1 vs. factor 2; factor 1 vs. factor 3; and factor 2 vs. factor 3).

In the first stage of our linear regression analyses, we include compensatory or catalyzing beliefs only, as predictors; at the next stage, we also include pro-environmental identity and PFC as predictor variables. In this, we mirror the approach used by Kaklamanou et al. (2015) who sought to assess the extent to which such beliefs were uniquely predictive of PEB (as opposed to only reflecting more general pro-environmental tendencies) while also considering the role of PFC. Given the previously observed relationship between spillover-related beliefs and pro-environmental identity, we also examine collinearity across these analyses; we do not find any evidence that this is problematic (VIF < 1.5 in all cases).

## Results

### Endorsement of Compensatory and Catalyzing Beliefs

As can be seen in Table 1, average levels of endorsement – where a participant stated they “entirely,” “mostly,” or “somewhat” agreed with the statement – varied from 15.9% (“It doesn’t matter how much energy I use when I’m at work or out of the house, as long as I try to be ‘green’ at home”) to 81.8% (“If I act in a manner that benefits the environment, it makes me more aware of other similar actions I can take”) across the full seven-country sample. Table 1 also shows the countries for which the lowest and highest levels of agreement were obtained.

**FIGURE 1:**
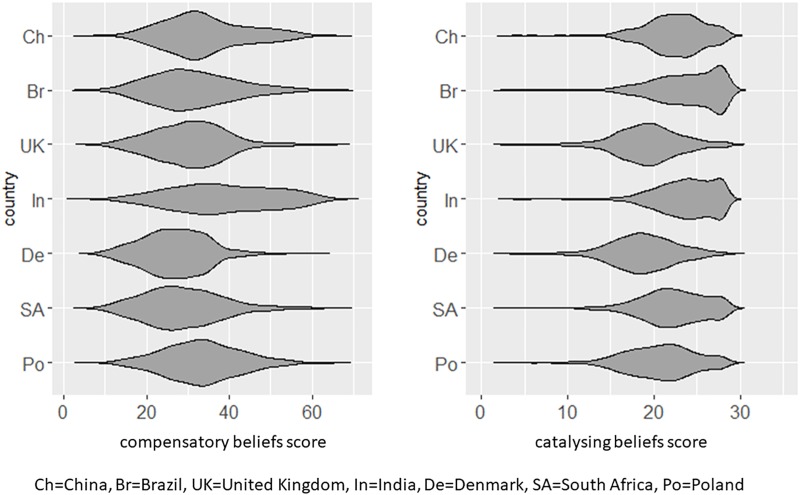
Distribution of Compensatory and Catalyzing beliefs scores across surveyed countries.

The overall distributional properties of both scales are shown in Figure 1. As can be seen here, relative to the other surveyed countries, responses are skewed and/or flattened in the case of India (compensatory beliefs), and Brazil and India (catalyzing beliefs).

### Relationship of Belief Types to Pro-environmental Identity and Preference for Consistency

Correlation tests were used to assess whether, and to what extent, pro-environmental identity predicts compensatory beliefs. This was undertaken separately for each of the seven countries. Table 3 shows Pearson’s *r* and *R*^2^ scores for the associations between pro-environmental identity and the compensatory beliefs scale across countries.

**Table 3:** Relationship between green identity and compensatory beliefs.

Country	Correlation (Pearson’s *r*)	*R*^2^
Brazil	−0.29^∗∗∗^	0.09
China	−0.44^∗∗∗^	0.19
Denmark	−0.41^∗∗∗^	0.16
India	−0.46^∗∗∗^	0.21
Poland	−0.23^∗∗∗^	0.05
South Africa	−0.39^∗∗∗^	0.15
United Kingdom	−0.42^∗∗∗^	0.17
Full dataset	−0.36^∗∗∗^	0.13

*^∗∗∗^p < 0.001.*

In all cases, the analysis supports the H1a prediction that identity and compensatory beliefs are inversely related. Pro-environmental identity explains between 5 and 21% of the variance in compensatory beliefs (adjusted *R*^2^ values), as shown in Table 3.

We carried out a similar set of correlation tests to assess whether, and to what extent, pro-environmental identity predicts catalyzing beliefs. In all cases, the analysis supports the H1b prediction that identity and catalyzing beliefs are positively related. Pro-environmental identity explains between 15 and 37% of the variance in catalyzing beliefs (adjusted *R*^2^ values), as shown in Table 4.

**Table 4:** Relationship between green identity and catalyzing beliefs.

Country	Correlation (Pearson’s *r*)	*R*^2^
Brazil	0.53^∗∗∗^	0.28
China	0.61^∗∗∗^	0.37
Denmark	0.49^∗∗∗^	0.24
India	0.39^∗∗∗^	0.15
Poland	0.53^∗∗∗^	0.28
South Africa	0.54^∗∗∗^	0.29
United Kingdom	0.60^∗∗∗^	0.36
Full dataset		0.27

*^∗∗∗^p < 0.001.*

We next carried out correlation tests to assess whether, and to what extent, PFC predicts compensatory beliefs. Table 5 shows results obtained.

**Table 5:** Relationship between PFC and compensatory beliefs.

Country	Correlation (Pearson’s *r*)	*R*^2^
Brazil	0.19^∗∗∗^	0.04
China	−0.04 (ns)	0.00
Denmark	0.02 (ns)	0.00
India	0.13^∗∗∗^	0.02
Poland	−0.06 (ns)	0.00
South Africa	0.02 (ns)	0.00
United Kingdom	0.04 (ns)	0.00
Full dataset	0.12^∗∗∗^	0.01

*^∗∗∗^p < 0.001.*

Our prediction of a negative relationship between these two constructs, H2a, was not supported. In only two of seven countries was a significant relationship obtained, and with only small amounts of variance explained.

Further analysis supports the prediction, H2b, that PFC and catalyzing beliefs are positively related. PFC explains between 7 and 28% of the variance in catalyzing beliefs (adjusted *R*^2^ values), as shown in Table 6.

**Table 6:** Relationship between PFC and catalyzing beliefs.

Country	Correlation (Pearson’s *r*)	*R*^2^
Brazil	0.26^∗∗∗^	0.07
China	0.53^∗∗∗^	0.28
Denmark	0.27^∗∗∗^	0.07
India	0.38^∗∗∗^	0.15
Poland	0.31^∗∗∗^	0.10
South Africa	0.34^∗∗∗^	0.11
United Kingdom	0.40^∗∗∗^	0.16
Full dataset	0.41^∗∗∗^	0.16

*^∗^p < 0.05, ^∗∗^p < 0.01, ^∗∗∗^p < 0.001.*

### Relationship Between Pro-environmental Behavior and Belief Types

We next assess the extent to which the different types of PEB described above are related to compensatory and catalyzing beliefs, using linear regression analyses.

As can be seen from Table 7, although a significant relationship is observed in all cases between compensatory beliefs and PEB, there is a divergence between the direction in which compensatory beliefs are predictive of PEB. In the case of resource/waste PEB and purchasing/food PEB the expected negative relationship is found; however, in the case of public-sphere PEB, a positive relationship is observed. Our hypothesis that compensatory beliefs would be inversely related to PEB, H3a, is therefore not supported.

**Table 7:** Relationships between PEB factors and compensatory beliefs.

	Dependent variable: public-sphere PEB (factor 1)			Dependent variable: resource/waste PEB (factor 2)			Dependent variable: purchasing/food (factor 3)		
	*B* (*SE*)	Beta	*R*^2^ (Δ*R*^2^)	*B* (*SE*)	Beta	Δ*R*^2^ (Δ*R*^2^)	*B* (*SE*)	Beta	Δ*R*^2^ (Δ*R*^2^)
Step 1			0.16			0.05			0.003
Compensatory beliefs	0.04 (0.001)	0.39^∗∗∗^		−0.02 (0.001)	−0.21^∗∗∗^		−0.005 (0.001)	−0.05^∗∗∗^	
Step 2			0.19 (0.04)			0.17 (0.12)			0.06 (0.06)
Compensatory beliefs	0.05 (0.001)	0.46^∗∗∗^		−0.01 (0.001)	−0.09^∗^		0.004 (0.001)	0.04^∗∗^	
Green identity	0.03 (0.002)	0.21^∗∗∗^		0.06 (0.002)	0.37^∗∗∗^		0.04 (0.002)	0.26^∗∗∗^	

*^∗^p < 0.05, ^∗∗^p < 0.01, ^∗∗∗^p < 0.001.*

In the case of the relationship between PEB and catalyzing beliefs (Table 8), our hypothesis, H3b, is more clearly supported: catalyzing beliefs are predictive of each of the three PEB types, and this relationship holds where pro-environmental identity is also included in the regressions. An unexpected negative relationship is observed between pro-environmental identity and one of the factors at Step 2. It is not clear why this result is obtained, given that pro-environmental identity is, on its own, positively associated with each factor. As we note above, we do not identify any concerns with collinearity in our regression analyses. Nevertheless, the relatively strong overall association between identity and catalyzing beliefs, as illustrated in Table 4, may indicate that this finding is an anomaly due to a relatively large degree of variance being shared between identity and catalyzing beliefs, in predicting PEB.

**Table 8:** Relationships between PEB factors and catalyzing beliefs.

	Dependent variable: public-sphere PEB (factor 1)			Dependent variable: resource/waste PEB (factor 2)			Dependent variable: purchasing/food (factor 3)		
	*B* (*SE*)	Beta	*R*^2^ (Δ*R*^2^)	*B* (*SE*)	Beta	Δ*R*^2^ (Δ*R*^2^)	*B* (*SE*)	Beta	Δ*R*^2^ (Δ*R*^2^)
Step 1			0.10			0.08			0.06
Catalyzing beliefs	0.07 (0.003)	0.31^∗∗∗^		0.06 (0.003)	0.28^∗∗∗^		0.06 (0.003)	0.24^∗∗∗^	
Step 2			0.11 (0.02)			0.17 (0.09)			0.08 (0.02)
Catalyzing beliefs	0.09 (0.003)	0.39^∗∗∗^		0.02 (0.003)	0.10^∗∗∗^		0.03 (0.003)	0.15^∗∗∗^	
Green identity	−0.02 (0.002)	−0.15^∗∗∗^		0.06 (0.002)	0.35^∗∗∗^		0.03 (0.002)	0.17^∗∗∗^	

*^∗∗∗^p < 0.001.*

As shown in Table 9, our analyses confirm our prediction, H4a, that compensatory beliefs are related to behavioral inconsistency. In each case, compensatory beliefs significantly and positively predict the degree of divergence between different types of PEB. The relationship is strongest for inconsistency between public-sphere and resource/waste PEBs.

**Table 9:** Relationships between PEB inconsistency and compensatory beliefs.

	Dependent variable: factor 1 vs. factor 2 scores			Dependent variable: factor 2 vs. factor 3 scores			Dependent variable: factor 1 vs. factor 3 scores		
	*B* (*SE*)	Beta	Δ*R*^2^ (Δ*R*^2^)	*B* (*SE*)	Beta	Δ*R*^2^ (Δ*R*^2^)	*B* (*SE*)	Beta	Δ*R*^2^ (Δ*R*^2^)
Step 1			0.11			0.02			0.04
Compensatory beliefs	0.03 (0.001)	0.33^∗∗∗^		0.01 (0.001)	0.12^∗∗∗^		0.02 (0.001)	0.19^∗∗∗^	
Step 2			0.11 (0.003)			0.03 (0.02)			0.04 (0.002)
Compensatory beliefs	0.03 (0.001)	0.31^∗∗∗^		0.01 (0.001)	0.08^∗∗∗^		0.02 (0.001)	0.20^∗∗∗^	
Green identity	−0.01 (0.002)	−0.06^∗∗∗^		−0.02 (0.002)	−0.13^∗∗∗^		0.004 (0.002)	0.03^∗^	
Pref. for consistency	0.00 (0.002)	−0.001 (ns)		−0.004 (0.002)	−0.03 (ns)		0.005 (0.002)	0.03^∗^	

*Factor 1, public-sphere PEB; factor 2, resource/waste PEB; factor 3, purchasing/food PEB. ^∗^p < 0.05, ^∗∗∗^p < 0.001.*

As shown in Table 10, our analyses do not support the prediction, H4b, that catalyzing beliefs are inversely related to behavioral inconsistency. We find a mix of divergent results here, as well as very low *R*^2^ values attributable to catalyzing beliefs, suggesting either a null or non-predicted relationship between these two variables.

**Table 10:** Relationships between PEB inconsistency and catalyzing beliefs.

	Dependent variable: factor 1 vs. factor 2 scores			Dependent variable: factor 2 vs. factor 3 scores			Dependent variable: factor 1 vs. factor 3 scores		
	*B* (*SE*)	Beta	Δ*R*^2^ (Δ*R*^2^)	*B* (*SE*)	Beta	Δ*R*^2^ (Δ*R*^2^)	*B* (*SE*)	Beta	Δ*R*^2^ (Δ*R*^2^)
Step 1			0.002			0.01			0.01
Catalyzing beliefs	0.01 (0.003)	0.04^∗∗^		−0.02 (0.003)	−0.10^∗∗∗^		0.02 (0.003)	0.09^∗∗∗^	
Step 2			0.05 (0.05)			0.02 (0.02)			0.02 (0.01)
Catalyzing beliefs	0.04 (0.003)	0.17^∗∗∗^		−0.003 (0.003)	−0.02 (ns)		0.03 (0.003)	0.14^∗∗∗^	
Green identity	−0.04 (0.002)	−0.26^∗∗∗^		−0.02 (0.002)	−0.15^∗∗∗^		−0.02 (0.002)	−0.11^∗∗∗^	
Pref. for consistency	0.003 (0.002)	0.02 (ns)		−0.001 (0.002)	−0.003 (ns)		0.001 (0.002)	0.03^∗^	

*Factor 1, public-sphere PEB; factor 2, resource/waste PEB; factor 3, purchasing/food PEB. ^∗^p < 0.05, ^∗∗^p < 0.01, ^∗∗∗^p < 0.001.*

## Discussion

The present study considers individuals’ beliefs in relation to how certain behaviors are thought of as triggering, justifying, or compensating for other behaviors. Our research is the most detailed exploration to date of the content, measurement, and relationships with other key indicators, of such spillover-related beliefs.

Our compensatory beliefs scale was found to have acceptable to good internal consistency (reliability) across the seven countries in which we were able to administer it; as did the 4-item catalyzing beliefs scale we devised. In the case of some specific measures used, we observed similar levels of endorsement as comparable previous research: for example, 16.2% of respondents in the Danish sample endorsed the view that reduced car use can compensate for flying on holiday, an identical figure to that obtained for an equivalent item used by Kaklamanou et al. (2015) with a United Kingdom sample. However, in contrast to previous research, for the most part we obtained substantially higher levels of agreement with the compensatory scale as a whole, as well as for specific items. We suggest there are two main reasons for this.

First, this was likely related to the use of items which did not exclusively affirm specific relations between predetermined behaviors or contexts. Whereas other research has tended to present specific behavior pairs in relation (or opposition) to one another, in the present study we also framed this in terms of more general statements. We also note the important caveat that the items used in the compensatory beliefs scale used some behavior-specific items, whereas the catalyzing beliefs scale used wording that reflected more general behavioral relations. This is likely to have influenced the overall higher levels of endorsement of the catalyzing beliefs scale, compared to the compensatory scale.

While we used several belief items that imply a more general relation between behaviors, in this, the statements we propose may well reflect an overlooked aspect of how compensatory beliefs operate in practice; rather than being rigidly tied to specific choices, a person’s beliefs may instead constitute an adaptable and generalized perspective on one’s own behavior in aggregate. This is in line with qualitative research by Hope et al. (2018), which argued that participants saw behavioral compensation on a cumulative and holistic level rather than in relation to distinct behavioral relations; these researchers likewise suggested that participant perspectives were at odds with survey items in which “single, predefined compensatory actions are pitted against one another.”

A second reason for the relatively higher levels of agreement with the compensatory scale used in the present study is likely to relate to our use of cross-national samples, and variability in country-level response distributions. While differences were not especially pronounced across the seven countries as a whole, it is noteworthy that Indian respondents in particular were more inclined to agree with these items, whereas those from Denmark were least likely to endorse them (Danish respondents were, indeed, also relatively less likely to endorse catalyzing beliefs). Some aspect of this is likely to relate to cross-cultural differences in survey responding, including the tendency for “acquiescent responding” (i.e., tendency to agree with statements) to vary cross-nationally (Johnson et al., 2005). It is worth noting in this regard that many of the seminal and influential studies of spillover have in fact been undertaken in Denmark (e.g., Thøgersen and Ölander, 2003; Lanzini and Thøgersen, 2014); which, from our research at least, would seem to comprise a population that is strongly inclined to reject compensatory beliefs.

Our use of a catalyzing belief scale revealed surprisingly high endorsement of the items proposed. While cross-country variability in patterns of responding is again evident – in particular, the scale distribution is skewed for the India and Brazil country samples – nevertheless participants across all countries appeared far more inclined to endorse catalyzing than compensatory beliefs. The wording of items could have reflected some aspect of people’s general pro-environmental attitudes or tendencies, as we note above, but it is of interest that the most-endorsed catalyzing beliefs item was one that most clearly presented the idea that one’s personal actions are linked in a positive manner. As with the compensatory beliefs scale, there may have been some sense in which these items were influenced by acquiescent responding, with this in turn varying on a cross-national basis. It is of note, however, that there does not appear to be a straightforward equivalence in responding by country, between the two belief types. In particular, whereas relatively high levels of agreement are found for this scale in Brazil and India, an equivalent pattern – whether in the same direction or inverse – is not shown for these countries for the compensatory beliefs scale.

We suggest that pursuing a deeper understanding of catalyzing beliefs – and similar constructs – offers a promising, and potentially constructive approach, to considering the ways in which people perceive their PEB as a whole. A large majority of people (around 90%) in Europe now report that they personally take action on climate change (Eurobarometer, 2017); where opportunities exist to make positive connections between such current, future, or recent action, particularly in relation to beliefs to which people widely subscribe, this could facilitate more widespread behavior change. The research literature already recognizes that there are multiple processes by which positive spillover can in principle occur – whether through a “foot in the door” approach (Thøgersen and Noblet, 2012), through self-identity (Van der Werff et al., 2014), or promoting self-efficacy (Lauren et al., 2016). However, our research suggests that one under-appreciated feature may be people’s own beliefs about the ways in which their own behaviors can be considered mutually reinforcing across choices and contexts.

In line with previous research, we have examined the extent to which spillover-related beliefs relate to pro-environmental (or “green”) identity, which is known to be both a precursor to action and relevant to behavioral spillover (Whitmarsh and O’Neill, 2010; Poortinga et al., 2013; Van der Werff et al., 2014; Nash et al., 2017). As in prior work, we also observe a negative association between identity and compensatory beliefs; conversely, we find a positive association between catalyzing beliefs and identity.

An advance offered through the present research, moreover, is an assessment of a link between our measures of spillover-related beliefs and PFC (Cialdini et al., 1995). In doing so, we consider whether these beliefs are correlated with a related and comparable construct which is not so straightforwardly associated with environmental concern and action. This enables us to assess the construct validity of spillover-related beliefs, in a way that has not previously been addressed.

We do observe a strong association between PFC and catalyzing beliefs, across the countries surveyed. This enables us to have some confidence in this novel measure, given that our view of catalyzing beliefs encompasses the idea of consistency across behaviors. Conversely, we do not find that PFC is inversely related to compensatory beliefs, as predicted. In this latter case, we speculate that where people subscribe to compensatory beliefs, this may not be as straightforwardly related to a lack of personal “consistency.” In particular, the characterizations of behavior across the compensatory items arguably do not preclude the idea of a logical pattern in one’s choices, albeit that this would be one that views one behavior as allowing for, or offsetting another. In this sense, to report that one favors “consistency,” as in the PFC items, may not be at odds with a view of behaviors counterbalancing each other.

We did observe a positive relationship between catalyzing beliefs and each of three types of PEB. However, our hypothesis that compensatory beliefs would inversely predict PEB was not supported. While this held in the case of private sphere (resource and waste) behavior, there was no clear or strong relationship with private sphere (purchasing and food) behavior and we unexpectedly observed a positive relationship with the cluster of public sphere behaviors, such as protesting or donating money.

One possible explanation for this may relate to the relatively high effort nature of the public sphere behaviors used, and their potential to allow a person to consider themselves to have “done their bit” had they carried them out. In line with a compensatory view, where people had taken such effortful action as contacting a politician or volunteering, this may be linked to feeling less obligated to take PEB in other areas. Although we did not anticipate such a finding, it would be in line with other research that has linked negative spillover to “single action bias” (Weber, 2010). Other work has found that people who carry out more private-sphere PEB may in turn be less inclined to offer support for environmental policy (Werfel, 2017); in the present research, our results hint at a relationship that might operate in the reverse direction also.

A direct assessment of how spillover-related beliefs might relate to behavioral (in)consistency was carried out in further analyses in the present study. This we argue is important to address, given that these spillover-related beliefs are, in essence, concerned with relations between behaviors as much as with PEBs in aggregate.

In support of our hypothesis, we observed a consistent finding across the three types of PEB, whereby endorsement of compensatory beliefs predicts inconsistency between different types of behaviors. The most pronounced effect observed was for inconsistency between public sphere behavior and private sphere resource/waste choices, suggesting that those holding compensatory beliefs are more likely to be inconsistent across these domains; this may be in terms either of relatively high levels of private sphere choices combined with lower levels of public sphere action, or vice versa.

We did not, however, find an association between catalyzing beliefs and behavioral (in)consistency; across the series of regressions carried out, this relationship was variously non-significant, negative, or positive. Moreover, the amount of variance explained by the catalyzing beliefs scale in these cases was relatively small, suggesting that this construct did not have a great deal of explanatory power here. One reason for this may be that the characterizations of behavior in the catalyzing beliefs scale would be more applicable across very similar types of behaviors, and rather less predictive of consistency between the distinct categories we assessed (e.g., in our case, between public sphere action and resource use behaviors). This would seem to be in line with the notion that spillover is more likely to occur between very similar types of behavior, than between ones perceived to be different (Littleford et al., 2014; Nash et al., 2017). Given the lack of a clear pattern here, we cannot in any case be confident that the catalyzing beliefs scale we developed is related to behavioral patterns, despite that we have found that it does convincingly predict overall levels of PEB. In relation to this, we recommend further developing the idea of “catalyzing” beliefs in more detail and depth, as this construct has received little attention outside the present study; as part of this, there may be opportunities to devise additional or complementary measures beyond the four items that we developed.

## Study Limitations and Future Research

The present study has obtained some support for the validity and reliability of spillover-related beliefs, as well as considering findings in the context of seven country samples. There are nevertheless some limitations to the research and areas for future development.

First, we are limited in our ability to make strong claims about the construct validity of the compensatory beliefs scale, given that this was not found to be related to PFC as expected. Nevertheless, compensatory items were found to predict both overall levels of behavior as well as behavioral inconsistency, suggesting their potential usefulness in future work. Conversely, while we did observe that catalyzing beliefs were related to PFC and overall levels of PEB – supporting the construct validity and predictive ability of this novel scale – this was nevertheless unrelated to behavioral (in)consistency. The lack of an association in this latter case raises questions over the ability of our novel catalyzing belief scale to explain patterns or linkages between behaviors, this being the aim of much spillover-related research.

We have considered the use of the compensatory and catalyzing beliefs measures in different cultural contexts, and observe some distinct differences in how people respond in these locations. It is not clear from our research whether this is linked to cross-cultural differences in response styles, fundamental differences in the extent to which people in different settings endorse such beliefs, or a combination of both. To date, there has been very little cross-cultural research concerning spillover and related topics, particularly outside of a developed country context. We therefore suggest that further attention is given as to whether these phenomena are generalizable and equivalent across different populations.

As in the case of much research in environmental psychology and related fields, we are limited by the use of self-report measures derived from a survey instrument. It would therefore be of value for these spillover-related beliefs to be tested in relation to observed behavior – and patterns of behavior – including in experimental contexts. In future research, it will be of value to link patterns of beliefs to more objective measures, such as home energy use or the recording of dietary choices.

Further testing and development of these types of measures in relation to comparable constructs would be valuable, in order to develop their validity. There are a range of theoretical models of relevance to behavioral consistency (e.g., see Mullen and Monin (2016) for an overview of approaches), which may have bearing on the ways in which people hold such beliefs, or are inclined to act upon them.

## Conclusion

The present study has progressed the understanding of spillover-related beliefs in several novel directions, providing one of the most detailed explorations to date of this topic area. Our research is, to our knowledge, the first to develop and assess a role for “catalyzing” beliefs, as well as considering those that are “compensatory.” In the case of both belief types, we have developed measures that portray generalized beliefs about patterns of behavior, in contrast to prior research which has relied on presenting linkages between specific types of action.

Our measures have been found to be reliable and to be associated with key psychological and behavioral measures, although our hypotheses were only partially supported in some cases: in particular, while we found support for our prediction that compensatory beliefs would be related to a lack of consistency between behavior types, the relationship was less straightforward in the case of catalyzing beliefs. The present research is the first, as far as we are aware, to consider spillover-related beliefs in the light of convergent constructs, through a comparison with a person’s preference for consistency and the degree to which they report (in)consistency across different types of behavior. We have also examined spillover-related beliefs for the first time in a cross-cultural context, including outside of a developed country setting. While we observe similar relationships between our key measures across cultures, divergence in the degree to which they are endorsed warrants further attention.

A priority for future research will be to assess how patterns of behavior and behavioral consistency are connected to spillover-related beliefs, as well as considering compensatory and catalyzing beliefs in more detail in the context of theoretically related constructs.

## Ethics Statement

This study was carried out in accordance with the recommendations of the British Psychological Society, with written informed consent from all subjects. All subjects gave written informed consent in accordance with the Declaration of Helsinki. The protocol was approved by the Ethics Committee, School of Psychology, Cardiff University.

## Author Contributions

The study was designed by SC, LW, and NN. The analysis was carried out by SC with assistance from LW. The manuscript writing was led by SC, with assistance and contributions from LW, NN, PH, and JL.

## Conflict of Interest Statement

The authors declare that the research was conducted in the absence of any commercial or financial relationships that could be construed as a potential conflict of interest.
